# Carbapenem-resistant gram-negative bacterial infection in intensive care unit patients: Antibiotic resistance analysis and predictive model development

**DOI:** 10.3389/fcimb.2023.1109418

**Published:** 2023-01-30

**Authors:** Qiuxia Liao, Zhi Feng, Hairong Lin, Ye Zhou, Jiandong Lin, Huichang Zhuo, Xiaoli Chen

**Affiliations:** ^1^ Department of Intensive Care Unit, First Affiliated Hospital of Fujian Medical University, Fuzhou, Fujian, China; ^2^ Department of Thoracic Surgery, First Affiliated Hospital of Fujian Medical University, Fuzhou, Fujian, China; ^3^ Department of Intensive Care Unit, National Regional Medical Center, Binhai Campus of the First Affiliated Hospital, Fujian Medical University, Fuzhou, China

**Keywords:** carbapenem-resistant gram-negative bacteria, risk factor, predictive model, logistic regression, area under the receiver operating characteristic curve

## Abstract

In this study, we analyzed the antibiotic resistance of carbapenem-resistant gram-negative bacteria (CR-GNB) in intensive care unit (ICU) patients and developed a predictive model. We retrospectively collected the data of patients with GNB infection admitted to the ICU of the First Affiliated Hospital of Fujian Medical University, who were then divided into a CR and a carbapenem-susceptible (CS) group for CR-GNB infection analysis. Patients admitted between December 1, 2017, and July 31, 2019, were assigned to the experimental cohort (n = 205), and their data were subjected to multivariate logistic regression analysis to identify independent risk factors for constructing the nomogram-based predictive model. Patients admitted between August 1, 2019, and September 1, 2020, were assigned to the validation cohort for validating the predictive model (n = 104). The Hosmer−Lemeshow test and receiver operating characteristic (ROC) curve analysis were used to validate the model’s performance. Overall, 309 patients with GNB infection were recruited. Of them, 97 and 212 were infected with CS-GNB and CR-GNB, respectively. Carbapenem-resistant *Klebsiella pneumoniae* (CRKP), carbapenem-resistant *Acinetobacter baumannii* (CRAB) and carbapenem-resistant *Pseudomonas aeruginosa* (CRPA) were the most prevalent CR-GNB. The multivariate logistic regression analysis results of the experimental cohort revealed that a history of combination antibiotic treatments (OR: 3.197, 95% CI: 1.561–6.549), hospital-acquired infection (OR: 3.563, 95% CI: 1.062–11.959) and mechanical ventilation ≥ 7 days (OR: 5.096, 95% CI: 1.865–13.923) were independent risk factors for CR-GNB infection, which were then used for nomogram construction. The model demonstrated a good fit of observed data (*p* = 0.999), with an area under the ROC curve (AUC) of 0.753 (95% CI: 0.685–0.820) and 0.718 (95% CI: 0.619–0.816) for the experimental and validation cohort, respectively. The decision curve analysis results suggested that the model has a high practical value for clinical practice. The Hosmer−Lemeshow test indicated a good fit of the model in the validation cohort (*p*-value, 0.278). Overall, our proposed predictive model exhibited a good predictive value in identifying patients at high risk of developing CR-GNB infection in the ICU and could be used to guide preventive and treatment measures.

## 1 Introduction

The widespread application and irrational use of carbapenem antibiotics have led to a steady rise in the incidence of carbapenem-resistant gram-negative bacterial (CR-GNB) infections (Nordmann and Poire, 2019) due to the presence of β-lactamase genes on mobile genetic elements (Logan and Weinstein, 2017). Thus, CR-GNB infections remain a major global public health concern requiring urgent prevention and control measures (Jean et al., 2022).

Carbapenem antibiotics are extensively used in patients admitted to the intensive care unit (ICU) because of the illness severity, low immunity and numerous invasive procedures performed. Consequently, the ICU has become a high-prevalence area of multi-antibiotic-resistant GNB infections in hospitals (Aleidan et al., 2021; Sader et al., 2022).

*Klebsiella pneumoniae*, *Acinetobacter baumannii* and *Pseudomonas aeruginosa* are the main organisms that may invade the respiratory tract, urinary tract, peritoneal cavity and bloodstream of patients to cause pneumonia, and urinary tract, peritoneal and bloodstream infections. These infections can considerably increase treatment costs and patients’ mortality risks (Brink, 2019; Wilke et al., 2022). In the absence of new and effective antibiotics, the strong antibiotic resistance of CR-GNB limits pharmacological options and exacerbates treatment difficulty (Garg et al., 2019).

Presently, inconsistent results have been reported in the few existing studies on predictive models of CR-GNB infections in ICU patients (Liang et al., 2022; Montrucchio et al., 2022). Therefore, to improve therapeutic outcomes, we believe it is essential to analyze CR-GNB antibiotic resistance to determine relevant risk factors and construct a reliable predictive model that could help identify high-risk patients and formulate individualized treatments. To this end, in this study, we retrospectively analyzed the data of patients with GNB infection in an ICU setting to provide a scientific basis for their clinical treatment, which could be used to strategize prevention and control measures for CR-GNB infection.

## 2 Patients and methods

### 2.1 Participants

This was a retrospective study on patients diagnosed with GNB infection and admitted to the ICU ward between December 1, 2017, and September 1, 2020, at the First Affiliated Hospital of Fujian Medical University (Fuzhou, China). The study inclusion criteria were: (1) age ≥18 years and (2) specimens had been collected after ICU admission for culture, from which a confirmed diagnosis of GNB infection and antibiotic susceptibility test results were obtained. Only the first positive culture result was selected in the case of multiple culture results. The exclusion criteria were as follows: (1) duration of ICU stay ≤ 24 h and (2) specimens sent for testing had been contaminated. The observation endpoint was patient discharge or in-hospital death.

The eligible patients were then classified into an experimental cohort and a validation cohort. The experimental cohort comprised patients admitted to the ICU between December 1, 2017, and July 31, 2019. They were divided into a CR-GNB group and a carbapenem susceptible (CS)-GNB group, and their data were subjected to multivariate logistic regression analysis for nomogram-based predictive model construction. Using the same selection and exclusion criteria, the validation cohort comprised patients admitted between 1 August 1, 2019, and September 1, 2020.

### 2.2 Definitions

CR-GNB: GNB identified from specimen culture that was resistant to imipenem, meropenem and ertapenem according to the antibiotic susceptibility test results (Ma et al., 2020).

Sepsis-related Organ Failure Assessment (SOFA) score: a six-organ dysfunction score of 0–24 points (Singer et al., 2016). Daily evaluations were performed based on the lowest score (Singer et al., 2016).

APACHE II score: the score comprised three components, namely the acute physiology score, age points, and chronic health points. It was used as a predictor of mortality rate in ICU patients (Naved et al., 2011).

Combination antibiotic treatment: the combined use of two or more types of antibiotics for anti-infective treatment.

Hospital-acquired infection: infection acquired after 48 h of hospital admission by a patient who had no infection and had not been in the incubation stage of infection at the time of hospital admission (Ministry of Health of the People's Republic of China, 2001).

### 2.3 Pathogenic bacteria and antibiotic susceptibility testing

Identification and routine antibiotic susceptibility testing of pathogenic bacteria were performed using the Vitek 2 Compact automated identification or antibiotic susceptibility testing system from bioMerieux (Lyon, France). Antibiotic susceptibility test results were interpreted using the 2017 edition of the Performance Standards for Antimicrobial Susceptibility Testing published by the Clinical and Laboratory Standards Institute (CLSI, 2017).

### 2.4 Data collection

The data retrieved from the patient’s medical records included sex, age, underlying diseases (such as hypertension, chronic obstructive pulmonary disease (COPD), malignancy, diabetes mellitus and cerebral infarction), hypoalbuminemia, history of recent hospitalization, glucocorticoid therapy, central venous catheterization, tracheal intubation, mechanical ventilation, hemodialysis, septic shock, history of antibiotic use within 1 month before the first positive culture test result, types of antibiotics used (including β-lactams, carbapenems, macrolides, tetracyclines, aminoglycosides, clindamycin, polypeptides, polymyxins, sulphonamides and quinolones), SOFA and Acute Physiology and Chronic Health Evaluation (APACHE II) scores on the day of admission, white blood cell (WBC) count, procalcitonin level, site of infection, pathogenic bacteria, and antibiotic susceptibility of bacteria.

### 2.5 Statistical analysis

Statistical analyses were performed using Stata SE15 from StataCorp LLC (Texas, United States of America). The normality of continuous variables was assessed using Shapiro–Wilk test. Non-normally distributed continuous variables are expressed as median (interquartile range) and compared using Mann−Whitney *U* test. Categorical data are expressed as count (frequency) and compared using the χ^2^ test. Fisher’s exact test was adopted when the expected frequency in the fourfold table was <5. All significant factors (*p <*0.05) in univariate analysis were used for multivariate analysis. Independent risk factors for CR-GNB infection were determined using multivariate logistic regression analysis, with variables selected using the stepwise forward selection method. Subsequently, a nomogram-based predictive model was constructed based on the independent risk factors. The model’s scoring criteria were established based on the magnitude of the regression coefficient of all independent variables to visualize the predictive model results. The model stability was assessed using the Hosmer−Lemeshow goodness-of-fit test, and the predictive ability of the model was evaluated using the area under the receiver operating characteristic (ROC) curve (AUC). The model’s practical value in clinical practice was determined using decision curve analysis (DCA). Differences were considered statistically significant when *p* was < 0.05.

### 2.6 Ethics approval and consent to participate

The study was approved by the Ethics Review Form for Medical Research and Clinical Technology Application and Ethics committee of the First Affiliated Hospital of Fujian Medical University (ID:[2015]084-1).

## 3 Results

### 3.1 Distribution of pathogenic bacteria and sites of infection

A total of 908 patients were admitted to the ICU ward of the First Affiliated Hospital of Fujian Medical University between December 1, 2017, and September 1, 2020. Based on the study criteria, 309 (experimental cohort, n=205; validation cohort, n=104) patients with GNB infection were eligible for this study. They comprised 244 men and 65 women, aged between 18 and 96 years. Of them, 97 had CS-GNB infection, and 212 had CR-GNB infection. Carbapenem-resistant *K. pneumoniae* (CRKP), carbapenem-resistant *A. baumannii* (CRAB) and carbapenem-resistant *P. aeruginosa* (CRPA) accounted for 131 (61.80%), 60 (28.30%) and 16 (7.55%) of the 212 detected strains in the patients, respectively, and were the most common GNB (**Table 1**). Overall, 96.70% of the pathogenic bacteria originated from the patients’ sputum (**Table 2**).

**Table 1:** Distribution of pathogenic bacteria associated with CR-GNB infection.

Pathogenic bacterium	No. of strains (n = 212)	Percentage (%)
*Klebsiella pneumoniae*	131	61.80
*Acinetobacter baumannii*	60	28.30
*Pseudomonas aeruginosa*	16	7.55
*Escherichia coli*	2	0.94
*Stenotrophomonas Maltophilia*	2	0.94
*Citrobacter freundii*	1	0.47

CR-GNB, carbapenem-resistant gram-negative bacteria.

**Table 2:** Sources of specimens in which pathogenic CR-GNB were detected.

Source of specimens	No. of strains (n = 212)	Percentage (%)
Sputum	205	96.70
Blood	3	1.42
Urine	0	0.00
Peritoneal cavity	2	0.94
Wound exudate	2	0.94

CR-GNB, carbapenem-resistant gram-negative bacteria.

### 3.2 Antibiotic susceptibility test results of CR-GNB main types

One hundred and thirty-one CRKP strains had low resistance to tigecycline and polymyxin but exhibited a resistance rate of ≥80% to all other antibiotics commonly used in clinical practice (**Table 3**). Sixty CRAB strains demonstrated a resistance rate of 65%, 70% and 80% to amikacin, trimethoprim-sulfamethoxazole and tobramycin, respectively. In addition, CRAB exhibited high susceptibility to tigecycline and polymyxin and a resistance rate of ≥90% to ticarcillin-clavulanic acid, cefoperazone-tazobactam, piperacillin-tazobactam, ceftazidime, ceftriaxone, cefepime, aztreonam, ciprofloxacin and levofloxacin. Piperacillin-tazobactam, ceftazidime, cefepime, amikacin, and polymyxin showed good *in vitro* anti-bacterial activity against the 16 CRPA strains. The resistance rate of the CRPA strains to tobramycin, ciprofloxacin, levofloxacin, aztreonam and trimethoprim-sulfamethoxazole was 50.00%, 56.25%, 56.25%, 81.25% and 100%, respectively (**Table 3**).

**Table 3:** Antibiotic susceptibility test results of the main types of CR-GNB.

Anti-bacterial agent	CRKP (n = 131)		CRAB (n = 60)		CRPA (n = 16)	
	No. of strains	Rate of resistance (%)	No. of strains	Rate of resistance (%)	No. of strains	Rate of resistance (%)
Ticarcillin/clavulanic acid	129	98.47	60	100	-	-
Cefoperazone-tazobactam	128	97.71	55	91.67	-	-
Piperacillin-tazobactam	128	97.71	59	98.33	4	25.00
Ceftazidime	130	99.24	59	98.33	5	31.25
Ceftriaxone	131	100.00	60	100.00	-	-
Cefepime	131	100.00	60	100.00	4	25.00
Aztreonam	130	99.24	60	100.00	13	81.25
Imipenem	131	100.00	60	100.00	16	100.00
Meropenem	131	100.00	60	100.00	16	100.00
Amikacin	105	80.15	39	65.00	2	12.50
Tobramycin	112	85.50	48	80.00	8	50.00
Ciprofloxacin	128	97.71	59	98.33	9	56.25
Levofloxacin	126	96.18	56	93.33	9	56.25
Trimethoprim-sulfamethoxazole	121	92.37	42	70.00	16	100.00
Tigecycline	11	8.40	1	1.67	-	-
Polymyxin	0	0.00	0	0.00	0	0.00

CR-GNB, carbapenem-resistant gram-negative bacteria; CRKP, carbapenem-resistant Klebsiella pneumoniae; CRAB, carbapenem-resistant Acinetobacter baumannii; CRPA, carbapenem-resistant Pseudomonas aeruginosa; -: susceptibility testing was not performed.

### 3.3 Comparison between the groups of patients in the experimental cohort

The experimental cohort consisted of 205 patients with GNB infection admitted to the ICU between December 1, 2017, and July 31, 2019. Of them, 62 had CS-GNB infection, and 143 had CR-GNB infection. Compared to patients with CS-GNB infection, those with CR-GNB infection had a significantly greater number of recent hospitalization, carbapenem use, central venous catheterization, and combination antibiotic treatment; number of types of antibiotics used; duration of antibiotic use and mechanical ventilation before the occurrence of infective bacteria; hospital-acquired infection; and mechanical ventilation ≥ 7 days (*p* < 0.05). In addition, patients with CR-GNB infection presented a significantly higher incidence of septic shock and in-hospital mortality than patients with CS-GNB infection (*p* < 0.05). No significant difference was observed in sex, age, history of hypertension, COPD, malignancy, diabetes mellitus, cerebral infarction, hypoalbuminemia, SOFA and APACHE II scores on the day of ICU admission, history of β-lactam use, tracheal intubation, hemodialysis, WBC count, neutrophil count, and procalcitonin level (*p* ≥ 0.05) between the two groups (**Table 4**).

**Table 4:** Comparison of baseline characteristics between the CS-GNB and CR-GNB groups of the experimental cohort.

Variable	CS-GNB(n = 62)	CR-GNB(n = 143)	*p*-value
Sex (male)	47 (75.8%)	110 (76.9%)	0.862
Age (years)	62.50 (54.00, 74.00)	62.00 (51.00, 74.00)	0.674
COPD	58 (93.5%)	129 (90.2%)	0.440
Hypertension	34 (54.8%)	77 (53.8%)	0.896
Malignancy	7 (11.3%)	20 (14.0%)	0.600
Diabetes mellitus	15 (24.2%)	38 (26.6%)	0.721
Cerebral infarction	16 (25.8%)	30 (21.0%)	0.447
Hypoalbuminaemia	16 (25.8%)	54 (37.8%)	0.097
History of recent hospitalization	40 (64.5%)	116 (81.1%)	0.010
History of carbapenem use	16 (25.8%)	77 (53.8%)	<0.001
History of β-lactam use	34 (54.8%)	94 (65.7%)	0.139
Combination antibiotic treatment	17 (27.4%)	86 (60.1%)	<0.001
Multi-site infection	13 (21.0%)	35 (24.5%)	0.586
Hospital-acquired infection	51(82.3%)	138(96.5%)	<0.001
History of glucocorticoid use	10 (16.1%)	47 (32.9%)	0.014
History of central venous catheterization	19 (30.6%)	83 (58.0%)	<0.001
Tracheal intubation	42 (67.7%)	94 (65.7%)	0.780
Haemodialysis	4 (6.5%)	14 (9.8%)	0.438
Mechanical ventilation ≥7 days	5 (8.2%)	53 (37.1%)	<0.001
Duration of mechanical ventilation before the occurrence of infective bacteria (days)	1.00 (0.00, 2.00)	3.00 (1.00, 8.00)	<0.001
SOFA score	5.00 (4.00, 6.00)	5.00 (4.00, 8.00)	0.119
APACHE II score	17.00 (13.00, 20.00)	17.00 (13.00, 21.00)	0.609
Duration of antibiotic use (days)	4.00 (1.00, 16.00)	12.00 (6.00, 22.00)	<0.001
No. of types of antibiotics used	1.00 (1.00, 3.00)	3.00 (1.00, 3.00)	<0.001
WBC count (10^9^/L)	11.51 (8.35, 15.45)	9.87 (7.44, 14.96)	0.213
Neutrophil count (10^9^/L)	9.79 (7.32, 13.39)	8.19 (5.68, 13.73)	0.190
PCT (ng/mL)*	0.39 (0.16, 2.62)	0.44 (0.15, 2.80)	0.949
Duration of hospital stay (days)	20.50 (17.00, 27.00)	19.00 (12.00, 26.00)	0.050
Duration of ICU stay (days)	19.50 (14.00, 24.00)	17.00 (11.00, 23.00)	0.033
Septic shock	12 (19.4%)	52 (36.4%)	0.016
In-hospital death	4 (6.5%)	24 (16.8%)	0.048

CS-GNB, carbapenem-susceptible gram-negative bacteria; CR-GNB, carbapenem-resistant gram-negative bacteria; COPD, chronic obstructive pulmonary disease; SOFA, sepsis-related organ failure assessment; APACHE II, acute physiology and chronic health evaluation; WBC, white blood cell; PCT, procalcitonin; ICU, intensive care unit. Continuous variables that did not follow a normal distribution are expressed as [M (P25, P75)]; categorical variables are expressed as [n (%)]. *Normal range for PCT was 0-0.06 ng/mL.

### 3.4 Risk factors for CR-GNB infection

The occurrence or non-occurrence of CR-GNB infection in ICU patients was set as the dependent variable. The variables with *p* < 0.05 in the univariate analysis (including history of recent hospitalization, combination antibiotic treatment, duration of antibiotic use, number of types of antibiotics used, hospital-acquired infection, history of glucocorticoid use, history of carbapenem use, history of central venous catheterization, duration of mechanical ventilation before the occurrence of infective bacteria, and mechanical ventilation ≥ 7 days) were used as independent variables. The multivariate logistic regression analysis was performed using the stepwise forward selection method to include single factors with *p* < 0.05 into the formula. The derived results indicated that combination antibiotic treatment (OR: 3.197, 95% CI: 1.561–6.549), hospital-acquired infection (OR: 3.563, 95% CI: 1.062–11.959) and duration of mechanical ventilation ≥ 7 days (OR: 5.096, 95% CI: 1.865–13.923) were independent risk factors for CR-GNB infection occurrence in ICU patients (**Table 5**).

**Table 5:** Univariate and multivariate analysis for factors associated with CR-GNB infection in ICU patients.

Variable	Univariate analysis			Multivariate analysis		
	OR	95% CI	*p-*value	OR	95% CI	*p-*value
History of recent hospitalization	2.363	1.212–4.608	0.012	-	-	-
Combination antibiotic treatment (X_1_)	3.994	2.084–7.656	<0.001	3.197	1.561–6.549	0.001
Duration of mechanical ventilation before the occurrence of infective bacteria (days)	1.050	1.018–1.083	0.002	-	-	-
No. of types of antibiotics used	1.544	1.222–1.950	<0.001	-	-	-
Hospital-acquired infection (X_2_)	5.953	1.972–17.970	0.002	3.563	1.062–11.959	0.040
History of glucocorticoid use	2.546	1.189–5.451	0.016	-	-	-
History of carbapenem use	3.354	1.739–6.470	<0.001	-	-	-
History of central venous catheterization	3.131	1.661–5.901	<0.001	-	-	-
Duration of mechanical ventilation (days)	1.165	1.067– 1.272	0.001	-	-	-
Duration of mechanical ventilation ≥7 days (X_3_)	6.471	2.437–17.181	<0.001	5.096	1.865–13.923	0.001

CR-GNB, carbapenem-resistant gram-negative bacteria; OR, odds ratio; CI, confidence interval; -: variables that were excluded through the stepwise forward regression method.

### 3.5 Nomogram-based predictive model of CR-GNB infection in ICU patients

**Figure 1:**
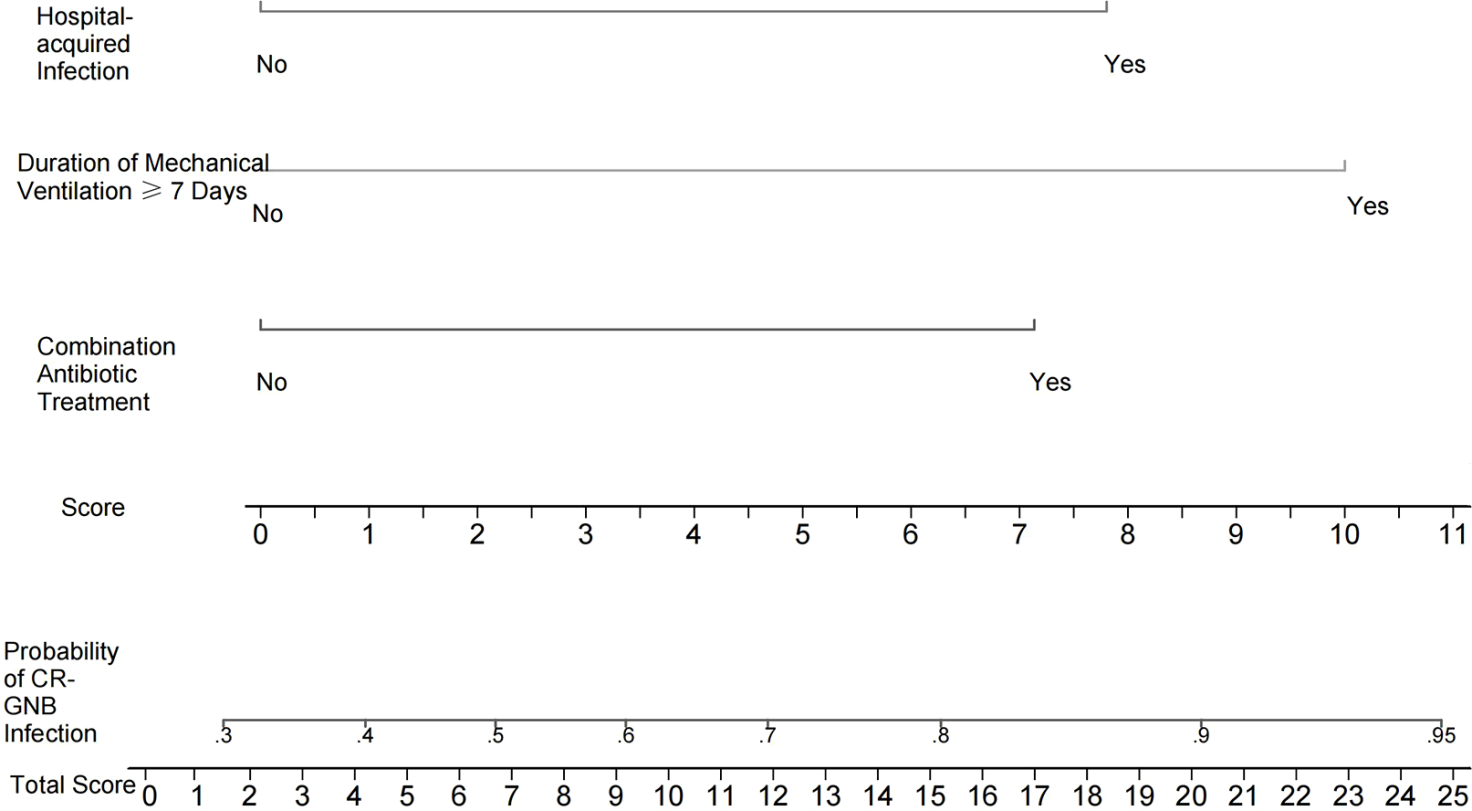
Nomogram for estimating the probability of CR-GNB infection in ICU patients. The scores of various independent variables were as follows: combination antibiotic treatment: 7.1 points, hospital-acquired infection: 7.8 points, and duration of mechanical ventilation ≥7 days: 10.0 points. The scores of the independent variables were summed to obtain the overall score, which can be used to predict the probability of CR-GNB infection in ICU patients. For instance, the predicted probability of CR-GNB infection occurrence in ICU patients was >80% when the total score was >16 points.

Independent risk factors screened from the multivariate logistic regression analysis were used to construct a nomogram-based predictive model of CR-GNB infection for ICU patients. The predictive model formula was *P* = 1/[1+exp [-(-1.090 + 1.162X_1_ + 1.271X_2_ + 1.628X_3_)]]. The scores of various independent variables were calculated using the corresponding regression coefficients and were as follows: combination antibiotic treatment: 7.1 points, hospital-acquired infection: 7.8 points, duration of mechanical ventilation ≥ 7 days: 10.0 points, and overall model score: 0–24.9 points. The scores of the independent variables were summed to obtain the overall score, which was used to predict the probability of CR-GNB infection of ICU patients. The plotted nomogram indicated that the predicted probability of the occurrence of CR-GNB in ICU patients was >80% when the total score was >16 points (**Figure 1**).

### 3.6 Internal validation of the predictive model

**Figure 2:**
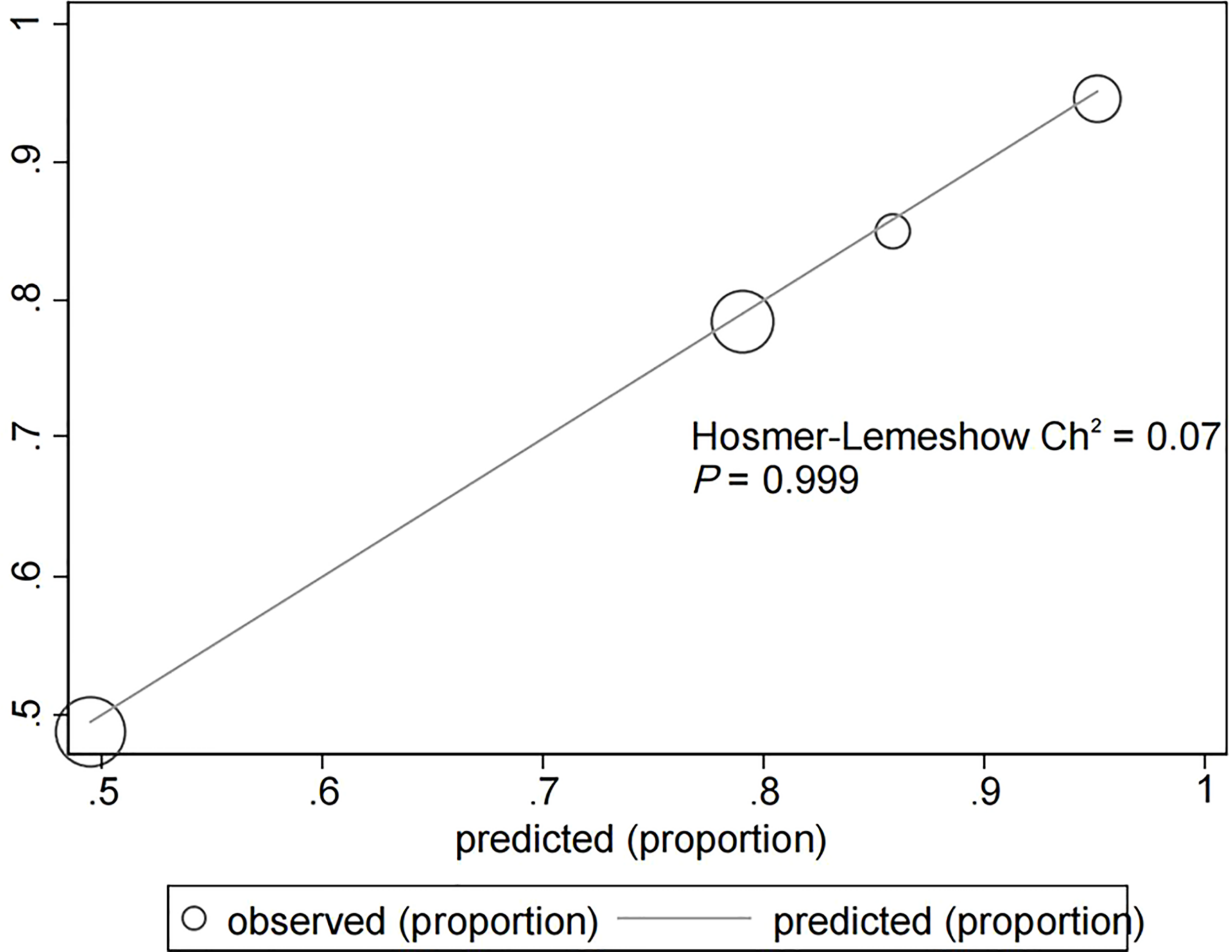
Hosmer−Lemeshow test provided a χ^2^ value of 0.07 (*p* = 0.999) for the predictive model in the experimental cohort, suggesting that the model presented a good fit for the observed data.

**Figure 3:**
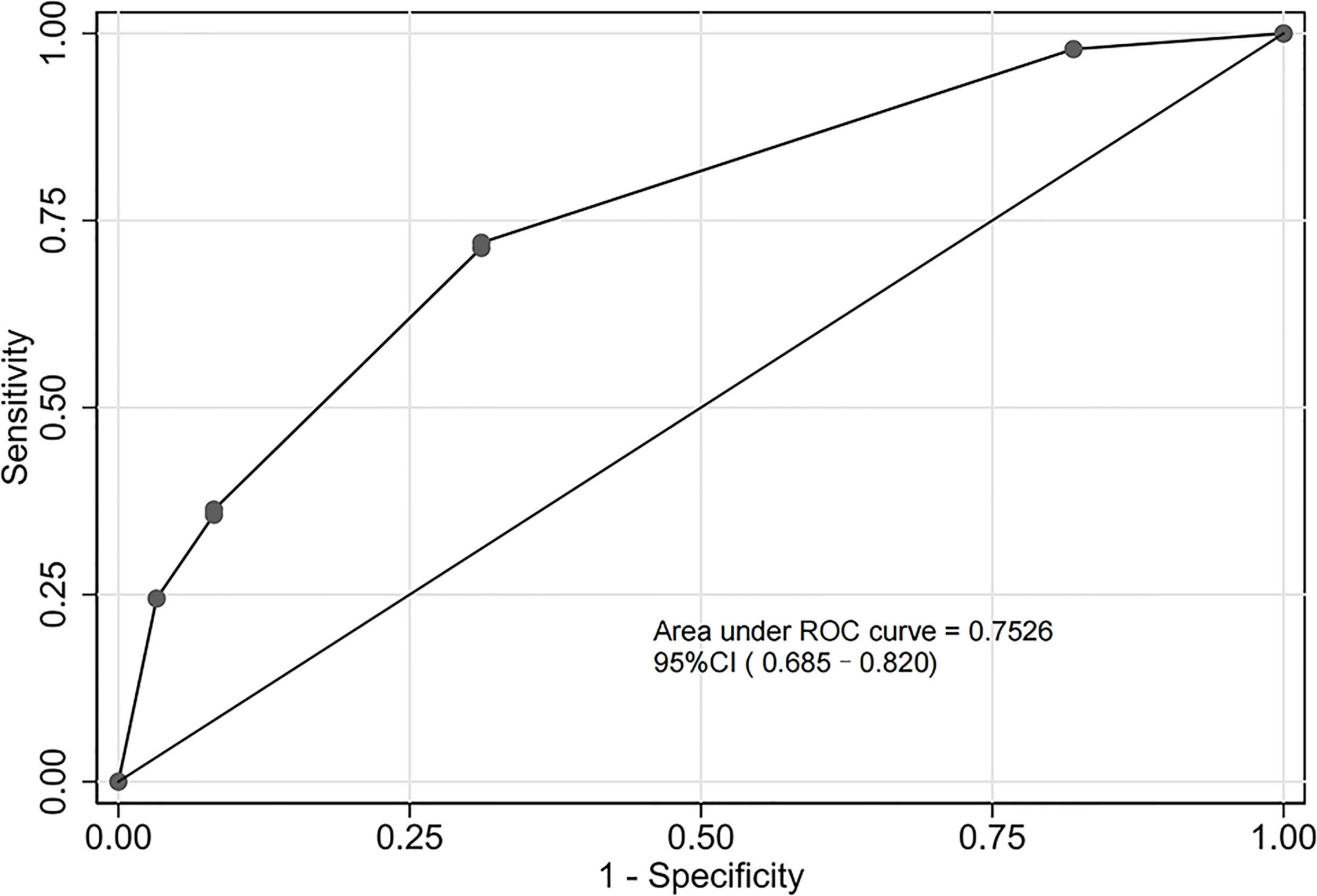
Receiver operating characteristic (ROC) curve of the predictive model in the experimental cohort. The predictive model had a good discriminative power with an area under the ROC curve of 0.753 (95% confidence interval: 0.685–0.820).

**Figure 4:**
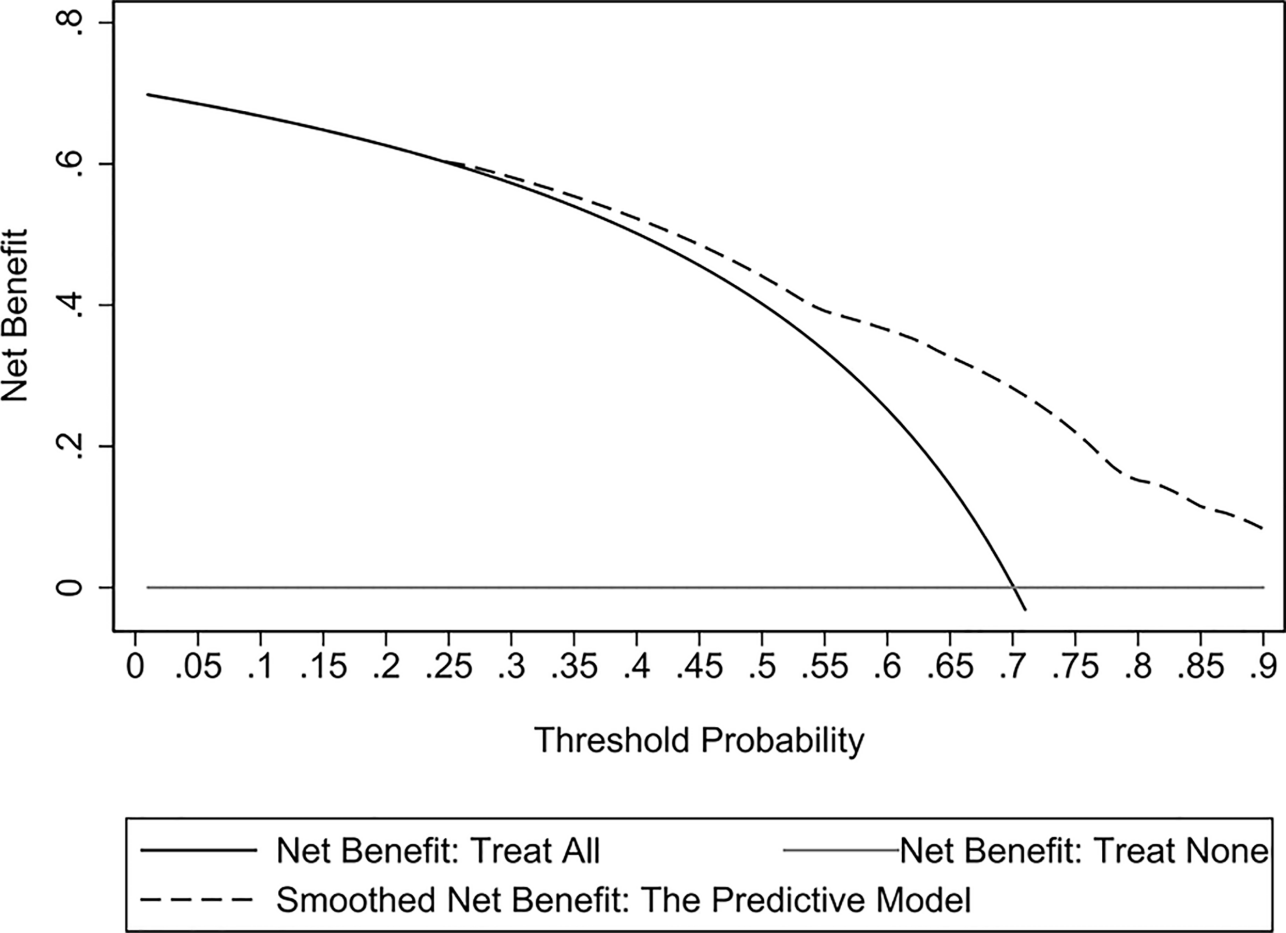
Decision curve analysis (DCA) curves of the predictive model in the experimental cohort. The DCA curve showed that the maximum clinical benefit could be obtained with the model when the predicted probability was >0.7.

The assessment of the goodness of fit of the model using the Hosmer−Lemeshow test showed an χ^2^ value of 0.07 and a *p*-value of 0.999, suggesting that the predicted probability of occurrence was consistent with actual probability and that the model presented a good fit of the observed data (**Figure 2**). The AUC of the predictive model was 0.753 (95% CI: 0.685–0.820), indicative of a good predictive performance (**Figure 3**). The AUC obtained using 1000 bootstrap replications was 0.753 (95% CI: 0.687–0.818), indicating that the model had good discriminatory power and repeatability. The DCA curve showed that the maximum clinical benefit could be obtained with the model when the predicted probability was >0.7, suggesting that the model could provide high practical value in clinical practice (**Figure 4**).

### 3.7 Validation of the proposed predictive model

**Figure 5:**
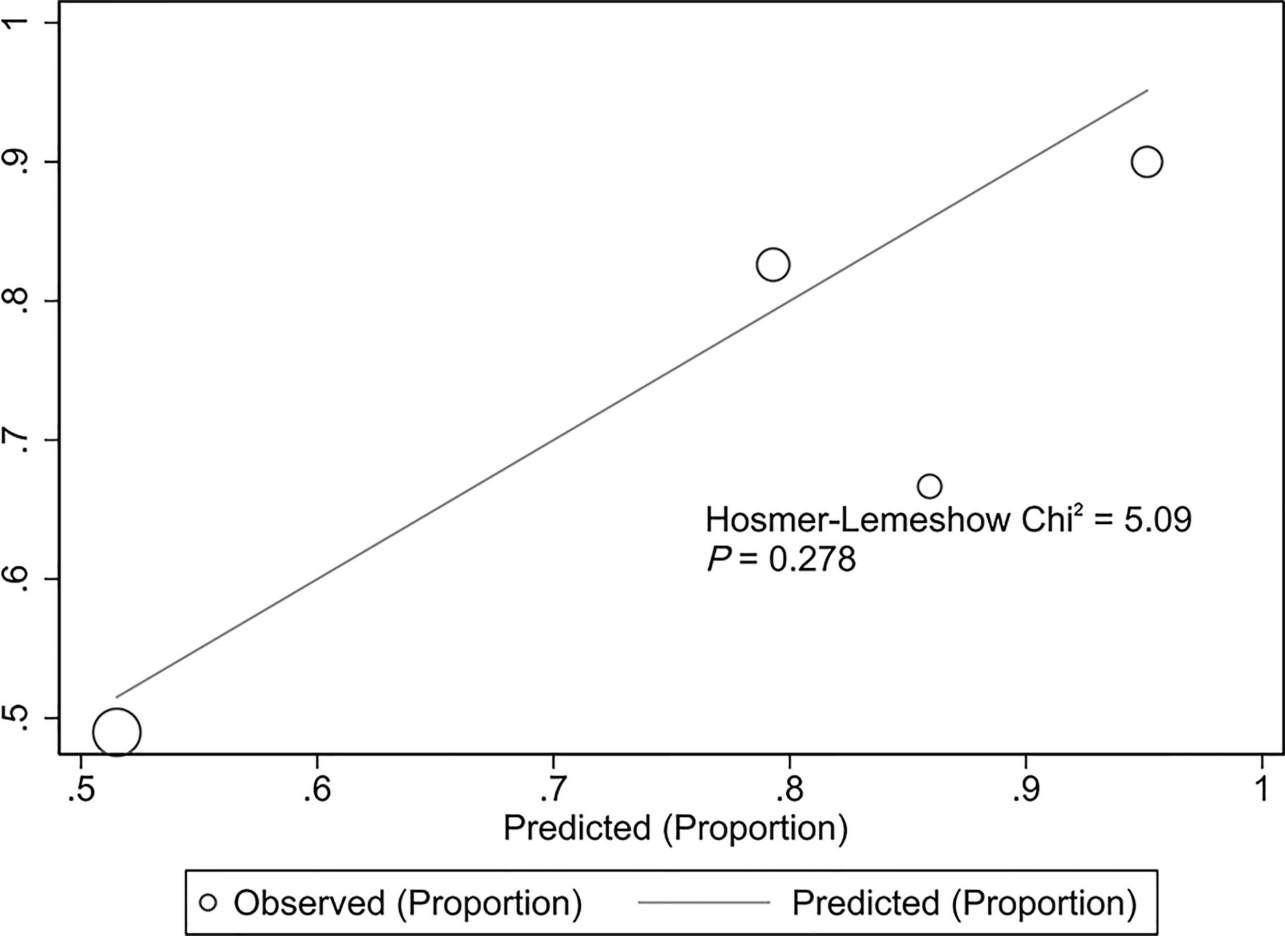
Hosmer−Lemeshow plot of the predictive model for the validation cohort. Assessment of the goodness of fit using the Hosmer−Lemeshow test showed an χ^2^ value of 5.09 and a *p*-value of 0.278, suggesting that the model presented a good fit in the validation cohort.

**Figure 6:**
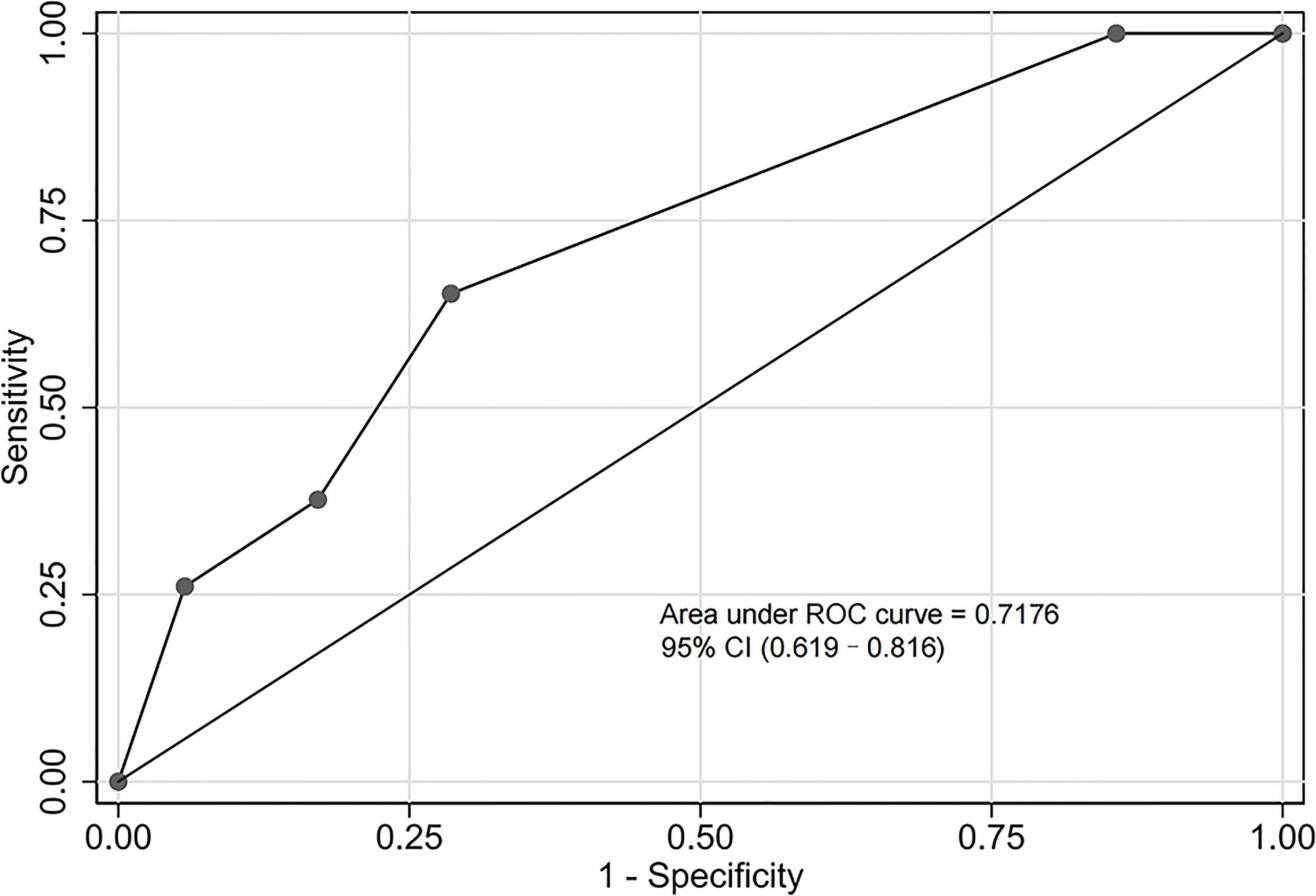
Receiver operating characteristic (ROC) curve of the predictive model for the validation cohort. The area under the ROC curve was 0.718 (95% confidence interval: 0.619–0.816), indicating a good prediction performance of the model in the validation cohort.

**Figure 7:**
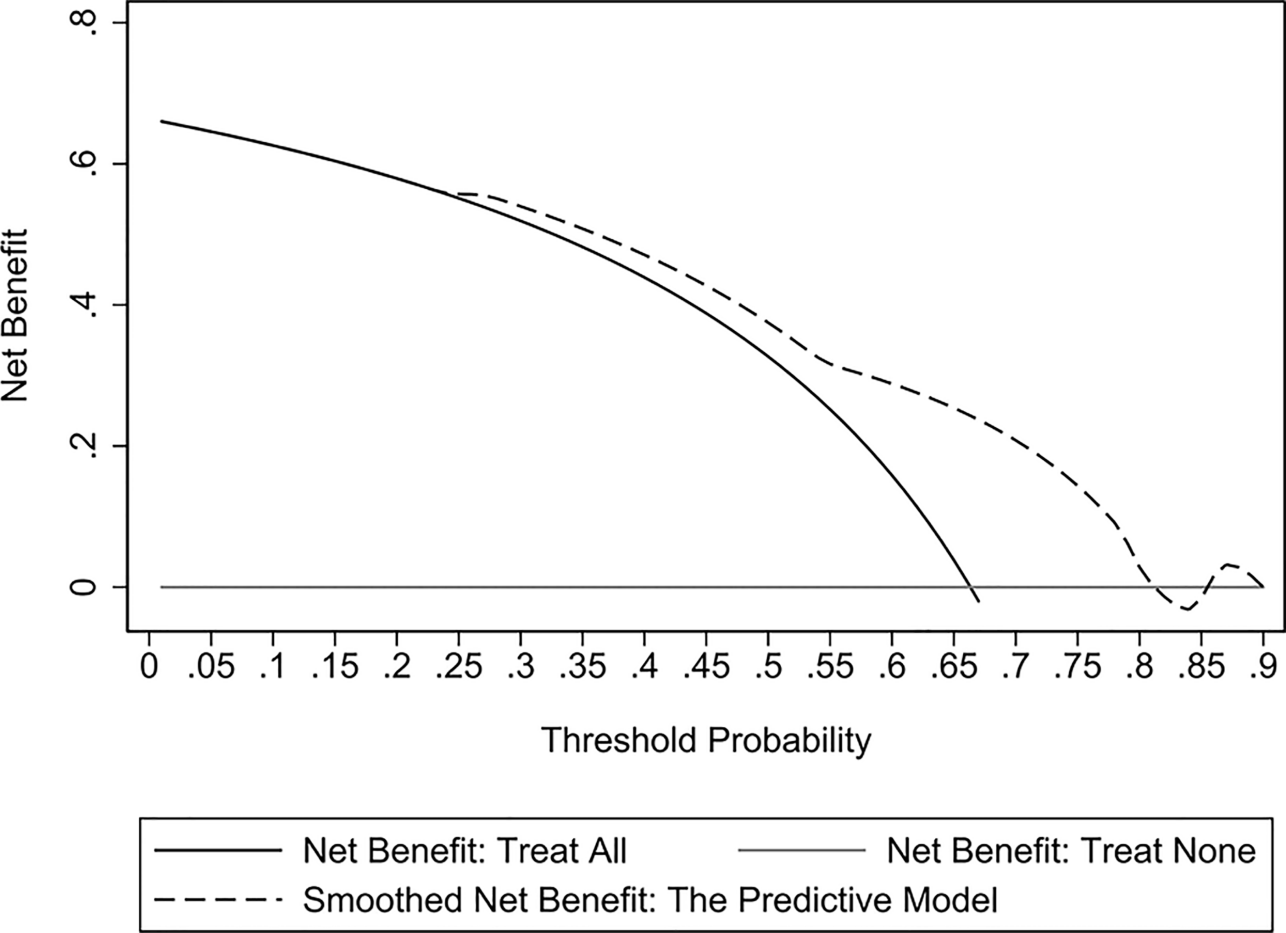
Decision curve analysis (DCA) curves of the predictive model for the validation cohort. The DCA curve showed that the model provided a high practical value in clinical practice when the predicted probability was > 0.75 in the validation cohort.

The validation cohort consisted of 104 GNB infection patients admitted to the ICU between August 1, 2019, and September 1, 2020. Of them, 35 had CS-GNB infection, and 69 had CR-GNB infection. The assessment of the goodness of fit using the Hosmer−Lemeshow test showed an χ^2^ value of 5.09 and a *p*-value of 0.278 (**Figure 5**), suggesting that the model presented a good fit in the validation cohort. The AUC was 0.718 (95% CI: 0.619–0.816), indicating a good prediction performance of the model in the validation cohort (**Figure 6**). The DCA curve indicated that the model showed a high practical value in clinical practice when the predicted probability was >0.75 in the validation cohort (**Figure 7**).

## 4 Discussion

The reported incidence rate of CR-GNB infection in the ICU is reported to range between 25.2% and 47% (Kiddee et al., 2018; Siwakoti et al., 2018). In addition, a substantial increase in the mortality of patients with CR-GNB infection has been observed in clinical practice (Dantas et al., 2019). In this study, we found that the probability of septic shock or in-hospital death was significantly higher in patients with CR-GNB infection than in patients with CS-GNB infection. Therefore, the early identification of patients with CR-GNB infection is an urgent need in clinical practice to improve their survival outcomes. This study retrospectively analyzed the antibiotic resistance of CR-GNB in infected ICU patients and successfully constructed and validated a clinical predictive model to assess the probability of CR-GNB infection occurrence in ICU patients. Altogether, our results could serve as a scientific basis to guide the clinical use of antibiotics and reduce irrational antibiotic use, which might contribute to improving patient outcomes and reducing hospitalization costs.

Our results indicated that *K. pneumoniae*, *A. baumannii* and *P. aeruginosa* were the three most prevalent CR-GNB in ICU patients, which is largely consistent with the findings of Ma et al. (Ma et al., 2020). In this study, *K. pneumoniae* had the highest proportion (61.80%) among all CR-GNB species detected. In addition, the 2021 results of the China Antimicrobial Surveillance Network (CHINET) surveillance of bacterial resistance across China revealed that the rate of *K. pneumoniae* resistance to imipenem and meropenem significantly increased from 3.0% and 2.9% in 2005 to 25.0% and 26.3% in 2018, respectively (Hu et al., 2022). In this study, we observed that CRKP in the specimens of our ICU patients had low resistance to tigecycline and polymyxin; however, it exhibited a resistance rate of ≥80% to all other antibiotics commonly used in clinical practice. This result indicates that the multi-antibiotic resistance of CRKP remains severe and poses major difficulties for clinical treatment, consistent with the findings of Zeng et al. (Zeng et al., 2021). A possible reason for this could be associated with the unique ability of *K. pneumoniae* to acquire exogenous resistance-encoding and hypervirulence-encoding genetic elements (Yang et al., 2021). The main mechanism by which *K. pneumoniae* develops carbapenem resistance involves the plasmid-mediated carbapenemase *K. pneumoniae* carbapenemase (KPC) (Qin et al., 2020), with *bla_KPC-2_* being the most common genotype in China (Li et al., 2019). Our results showed that CRKP, CRAB and CRPA had high susceptibility to polymyxin, suggesting that polymyxin combined with other antibiotics could achieve good therapeutic effects in treating CR-GNB infections.

We found that combination antibiotic treatment was an independent risk factor for CR-GNB infection in ICU patients. Previous research has also demonstrated a relationship between the history of antibiotic use and CR-GNB infection (Li et al., 2019; Qin et al., 2020), which might be attributed to the fact that combination antibiotic treatment causes dysbiosis in the microbiota, whereby the majority of susceptible bacteria are eradicated, while the antibiotic-resistant strains survive. In this regard, using comparative genomics to investigate the population dynamics of *Acinetobacter baumannii* during host colonization, Wen et al. found that antibiotic usage and host environment could selectively drive the rapid adaptive evolution to enhance their colonization (Wen et al., 2014). Additionally, Wang et al. found a novel *Pseudomonas aeruginosa* strain that underwent rapid adaptive evolution during ventilator-associated pneumonia to become resistant to β-lactams due to the selective pressure imposed by intensive β-lactam treatments (Wang et al., 2017). Thus, antibiotic resistance of bacteria changes with time and the excessive use of antibiotics may accelerate this process (Raman et al., 2018), thereby increasing the risks of CR-GNB infection. Therefore, the occurrence of CR-GNB infection could be effectively reduced through the minimization of irrational antibiotic use and timely identification of pathogenic bacteria for targeted anti-infective treatment. Our results suggest that hospital-acquired infection may also increase the risk of CR-GNB infection in ICU patients. Given that multiantibiotic-resistant bacteria survive for long periods in hospital environments and the low immunity of inpatients, inadequate or inappropriate prevention and control measures by a hospital may lead to a high tendency of cross-infection or even an outbreak among healthcare workers and patients. O’Hara et al. reported that the gloves and gowns of healthcare workers were prone to contamination by carbapenem-resistant Enterobacterales members and are considered to play an important role in the transmission of antibiotic-resistant bacteria among inpatients (O'Hara et al., 2021). This finding suggests that improvements in hand hygiene and environmental disinfection for reducing the occurrence of hospital-acquired infection have effectively prevented CR-GNB infection. Our results also indicated that the duration of mechanical ventilation ≥7 days was associated with CR-GNB infection in ICU patients, which was similar to the results of previous studies (Liu et al., 2018; Palacios-Baena et al., 2021). These findings suggest that for patients receiving mechanical ventilation, the possibility of weaning should be evaluated daily, and timely measures should be implemented to reduce CR-GNB infection.

Recently, several predictive models have been proposed for subsequent infections after CR-GNB colonization in different types of diseases. In the study of Wu et al. (Wu et al., 2022), after reviewing the data of patients with hematological malignancy (n = 437), they constructed a scoring model based on mucositis, duration of agranulocytosis, hypoalbuminemia and remission induction chemotherapy, which could stratify high-risk from low-risk patients with an OR (95%CI) of 3.347 (2.218-5.094) and AUC of 0.708. However, they did not investigate the different strains of CR-GNB nor validate their findings. Yan et al. constructed a model for the early prediction of death risk in severe acute pancreatitis patients infected with GNB (Yan et al., 2022). They found that platelets, hemoglobin, septic shock and carbapenem resistance were independent risk factors for mortality in these patients, based on which they proposed a nomogram for predicting the risk of mortality. Their model demonstrated good reliability based on an AUC of 0.942 and 0.911 in their training and validation cohort. However, despite these promising findings, most existing models either lacked validation or focused on only one disease. Comparatively, our proposed model was based on different strains of CR-GNB infection (**Table 1**), assessed the different specimens (**Table 2**), performed antibiotic susceptibility tests for a wide range of commonly used anti-bacterial agents (**Table 3**), and involved severely diseased patients with different ailments (**Table 4**). Our multivariate logistic regression analysis revealed that a history of combination antibiotic treatment, hospital-acquired infection and duration of mechanical ventilation ≥7 days were independent risk factors for CR-GNB infection in ICU patients. Subsequently, a nomogram-based predictive model was constructed based on these three risk factors, successfully validated (AUC, 0.718), and demonstrated good repeatability of the model. Model scores can be used to predict the probability of CR-GNB infection occurrence in ICU patients, whereby patients at high risk of CR-GNB infection could be subjected to early intervention, and excessive intervention could be avoided for those at low risk of CR-GNB infection. Our proposed model has a certain level of novelty, possesses promising practical implications, and its use might improve the clinical decision-making process of clinicians to increase the net benefits of treatments and positively improve patient outcomes.

Despite the interesting findings reported here, there were some limitations that should be mentioned. First, since this was a single-center retrospective study with a small number of patients and considering the smaller number of cases in the validation cohort, further prospective large-sample studies are needed to validate the effects of the predictive model on a wider range of patients and the clinical decisions of clinicians. Second, we only selected the first positive culture and did not account for cases such as second episodes of ventilator-associated pneumonia caused by CR-PsA or CRAB, multiple culture results, etc., which should be considered in future studies to improve the proposed nomogram. Third, we did not completely exclude the effects of collinearity in the effects of carbapenem for combination use of antibiotics because there were patients who did not use carbapenem either as single or combination therapy, thereby portraying real-world clinical scenarios to a certain extent. Fourth, since all carbapenem-resistant gram-negative bacteria were resistant to imipenem and meropenem, no analysis for resistance to imipenem and sensitivity to meropenem was performed. Lastly, the inclusion of pan-carbapenem-resistant bacteria could be an improvement for future studies to formulate a more widely applicable nomogram.

## 5 Conclusion

In conclusion, this study analyzed the antibiotic resistance of CR-GNB. Our findings revealed that CRKP, CRAB, and CRPA were the three most prevalent CR-GNB in ICU patients. Based on independent factors associated with CR-GNB infection, we constructed and successfully validated a nomogram for CR-GNB infection risk prediction of ICU patients. Altogether, these findings could be used to formulate individualized treatments for these patients, based on which effective control and preventive measures could be taken to reduce the risk of CR-GNB infection, especially in ICU settings.

## Data availability statement

The raw data supporting the conclusions of this article will be made available by the authors, without undue reservation.

## Ethics statement

The studies involving human participants were reviewed and approved by the Ethics Review Form for Medical Research and Clinical Technology Application and Ethics Committee of the First Affiliated Hospital of Fujian Medical University. The patients/participants provided their written informed consent to participate in this study.

## Author contributions

QL, XC, and ZF conceived and designed the study. QL, ZF, YZ, and HL collected the data. XC, HZ, QL, and ZF performed the analysis and interpreted the results. QL, HZ, XC, and ZF drafted the manuscript. QL, ZF, HZ, JL, and XC revised the manuscript. HZ and XC supervised the study and manuscript submission. All authors contributed to the article and approved the submitted version.
